# Aneurism of the Common Carotid Artery, Being an Analysis of Seventeen Cases of This Disease

**Published:** 1843-09

**Authors:** Robert S. Wendel

**Affiliations:** Murfreesboro, Tennessee


### WESTERN JOURNAL

OF

### MEDICINE AND SURGERY

---

SEPTEMBER, 1843

---

ART. I.—*Aneurism of the Common Carotid Artery, being an Analysis of Seventeen Cases of this disease.* By ROBERT S. WENDEL, M.D., of Murfreesboro, Tennessee.*

Aneurism, in whatever part of the body occurring, has always been considered an interesting disease to the surgeon, and a dangerous one to the patient. The opinions of the ancients relative to this disease were both vague and unsatisfac-

---

* We regret that our limits do not permit us to insert the whole of this excellent paper, embracing, as it does, the most elaborate account of the nature and treatment of aneurism of the primitive carotid artery with which we are acquainted. It occupies sixty-eight pages of manuscript, and forms the Inuagural Dissertation which Mr. Wendel submitted, last spring, to the President, Managers and Faculty of the Louisville Medical Institute, for the degree of Doctor of Medicine. The essay is illustrated by beautiful diagrams, and reflects much credit upon the author.—EDS.

tory, and to no artery is this remark more applicable than to the carotid. Ignorant of the anatomy and physiology of the brain, and of the arteries supplying it with blood, aneurisms of the carotid were permitted to take their own course, it being not even pretended to ligate that vessel for fear of injuring the functions of that important organ. "A case is given by John Bell, in which, so late as the year 1807 or 1808, a poor woman was suffered to perish by hemorrhage from the sac under the eyes of himself and colleagues, a whole college of surgeons, at that particular period, claiming to be surgeons to the Royal Infirmary of Edinburgh."†

That a ligature applied to this vessel will not injure the brain has long since been proved, and even both carotids have been tied in the human subject, with but a short period of time intervening, without any ill effects arising.

Cases are also recorded in which, on post-mortem examination, one or both carotids were totally obstructed, without any impairment of the intellect having been noticed during life.

The first instance in which the carotid was tied is mentioned in Benjamin Bell's Surgery. The operation was performed on account of a wound in extirpating a tumour. Mr. Abernethy is said to have tied it next, and after him, Mr. Fleming, a naval surgeon, in 1803; in both cases for wounds.

Sir Astley Cooper, however, was the first who tied this vessel for aneurism, but the case was unsuccessful. The ligation of the carotid may now be considered an every-day operation, being frequently performed for wounds, aneurisms, and as a preliminary measure in the extirpation of tumours about the face; though this latter has now partly fallen into disuse.

The carotid is not so often affected with aneurism as is generally supposed. Out of sixty-three cases of the disease reported by Mr. Hodgson, this artery was affected in only two instances, both occurring in males, while of the ascend-

* Liston's Operative Surgery.

ing aorta, arteria innominata, and aortic arch, there were twenty-one cases, descending aorta eight, subclavian and axillary five, inguinal and popliteal fifteen. Of these sixty-three cases only seven occurred in females.

Out of one hundred and seventy-nine cases drawn up by Mons. Lisfranc, exclusive of those of the aorta, there were seventeen cases in which the carotid was affected.

*Diagnosis.*—Aneurisms of the carotid artery are liable to be confounded with other diseases. They may be mistaken for scrofulous enlargement of the lymphatic ganglions, encysted tumors, goitre and dilatation of the internal jugular vein.

The lymphatic ganglious, situated along the antero-lateral aspect of the neck, often become enlarged, and receiving the impulse of the carotid artery, may simulate aneurism of that vessel. The disease generally occurs in young subjects, and is attended by all the marks of a scrofulous diathesis, such as a fair skin, light hair and eyes, tumid lips and abdomen, and precocious intellect. The tumor is hard to the touch, inelastic, irregularly lobulated, and commonly longer in the vertical than in the horizontal direction.

It is easily influenced in its volume and consistence, by exposure to cold, and by the state of the constitution.

In the early stage of the disease, it is moveable and may be lifted away from the surrounding parts; by and by, however, it contracts adhesions, and becomes fixed in its situation. In many cases it is disposed to run into suppuration; the skin assumes a livid or purplish appearance, and the tumour at length yields at one or more points, discharging a thin yellowish matter intermixed with curdy flakes.

Hydrocele of the neck may also be mistaken for this disease. "The characters, however, by which the former may be distinguished from other affections, are, absence of pain, and tenderness on pressure, slight fluctuation, the slow progress of the tumour, years generally elapsing before it attains much development, and, above all, the history of the case. When the tumour projects outwardly over the carotid artery it might be mistaken for aneurism, from which, however, it may, in general, be readily discriminated, by the elevation of the entire swelling from the impulse of the blood, and by the want of that alternate expansion, and retrocession, which are present in genuine aneurism."*

Aneurism of the carotid artery cannot always be easily distinguished from bronchocele or goitre. In the latter disease the tumour is comparatively insensible, devoid of pulsation, irregular in its consistence, being soft at some points, and hard at others, and more or less moveable. During deglutition, it accompanies the motions of the trachea and larynx. It is more common in the female than in the male, and is almost always very tardy in its growth, years elapsing before it attains much volume. Aneurism, on the contrary, progresses comparatively rapidly, the swelling is of pretty uniform consistence, does not follow the movements of the wind-pipe, imparts a distinct pulsation to the finger, and may be emptied of its contents by pressure along the course of the vessel, in which it is situated. When the tumour is large, the beating is generally distinguishable at the distance of a few feet from the patient, and if the ear be applied to the surface of the aneurism, the blood is heard to rush into it with a peculiar whizzing noise. In most instances of aneurism, also, the vessel is deeper seated than goitre, and the pulsation is so strong as scarcely to be mistaken.

When goitre, however, is extensive, and the pulsations of the carotid are freely communicated to it, the diagnosis will be difficult, if not impossible. A case of this kind, in which some of the most able practitioners of Philadelphia were deceived, is related by the late Dr. Griffiths in the ninth volume of the Eclectic Repository. The tumour was situated directly over the course of the carotid, and was pronounced by Dr. Chapman, Dr. Morgan, and the late Dr. Dorsey, to be of an aneurismal nature. The patient at length died, and upon dissection, Dr. Parrish ascertained that the vessel in

---

* Gross' Liston.

question was perfectly sound, and that the tumour was composed entirely of the thyroid gland. The neck was very short, the pulsation of the artery was imparted to the swelling, there was even a strong resemblance to the aneurismal thrill, and the morbid growth adhered so firmly to the surrounding parts that it could not be drawn away from them.

Another case, quite as remarkable, occurred some years ago in Paris. A creole negro had a tumour in the neck, which was submitted to the inspection of nearly all the distinguished surgeons of that metropolis, who pronounced the disease an aneurism of the carotid artery. The patient afterwards died of some other malady, when it was ascertained by Boyer that no such affection had existed, but simply an extensive enlargement of the lymphatic ganglions of the neck.

Aneurism of the carotid artery may also be mistaken for dilatation of the internal jugular vein. The latter, however, is a very rare affection, and the tumour is generally situated very low in the cervical region, appearing just above the sternum. It is also softer and more compressible than a true aneurismal swelling; the pulsation is more feeble and tremulous, the dilatation disappears when pressure is applied to the upper part of the vessel, and the turgescence is more diffused in its diameter, reaching sometime as high nearly as the angle of the jaw.

Aneurisms of the arch of the aorta are also liable to be confounded with this disease. "Aneurisms of long standing, which no longer throb, and the integuments over which are changed in color and likely to burst, may easily be mistaken for chronic abscesses, as the neck is frequently the seat of such diseases."*

Whenever any doubt exists as to the true nature of a tumour it may at once be dispelled by the introduction of a cataract needle or a delicate trocar.

*Spontaneous cure.*—Aneurisms of the carotid artery, like

---

* Cooper's Surgical Dictionary.

the same disease in other parts of the body occasionally undergoes a spontaneous cure. This, however, is of rare occurrence. The manner in which a spontaneous cure may take place is thus described by Mr. Wardrop.

1st. By the aneurismal sac being so strengthened and filled with coagulable lymph, that no fluid blood can pass into it, and of course all danger of rupture is prevented, whilst at the same time the original canal of the vessel remains pervious and carries on the circulation.

2d. By not only the sac being filled with coagulable lymph, but by the arterial canal being also obliterated.

3d. By the tumour acquiring such a size and position, that by pressing on the trunk of the artery, above or below, the sides of the aneurism are thus brought into contact and adhere.

4th. A spontaneous cure of aneurism has also been known to take place where the whole circumference of the vessel has been dilated, the sac being filled up with coagulum, but leaving a canal in the midst of the tumour through which the blood continued to pass.

5th. By a process of suppuration taking place in the sac after both it and the artery, both above and below the tumour, have been filled with coagulum. In such a case the integuments ulcerate, and a great quantity of the coagulum is discharged through the opening, which must be removed by the process of absorption.

The common tendency, however, of the disease is to a fatal termination, and the patient should therefore be placed under treatment, as soon as the nature of the lesion is ascertained.

The Hunterian operation was performed in nine cases. Of these, four occurred in females, and but three, or just one-third, terminated fatally. Of the latter, one died on the twenty-first day from inflammation of the sac, which extended to the base of the brain; one on the sixty-seventh from inflammation and repeated hemorrhages; and in one, that of Mr. Cline, no dissection is given.

In six of the cases, the disease occurred on the right side, in one on the left, in one on both sides (though the artery of but one side was tied) and in one, the situation is not given.

The occupation of the patients is given in only five of the cases; of these, two were laborers, one a porter, one a sportsman, and one a prostitute.

The cause of the disease is stated in but four. In two, it was brought on by a fall from a horse; in one, it was noticed soon after the man carried a heavy load on his back, and in one it was produced by a wound.

The duration of the disease, prior to the ligation of the artery, is noticed in all of the cases, except three. In one, it had existed six months, in one three months and a half, in one eleven months, in one eleven days, in one three months, in one three years.

The volume of the tumour, in four, was *very large*, in one five inches in length, by four in depth, in one of the size of a large walnut, in one the size of a walnut, in one of an egg, and in one as large as a goose egg.

The length of time required in performing the operation is not stated in a single instance.

In three of the cases two ligatures were used, and in one of these, the artery was divided between them, as recommended by Mr. Abernethy. In this case there was a difference of forty-eight hours in the separation of the ligatures, the upper coming away on the fifteenth day of the operation, the lower on the seventeenth.

In the remaining two cases both ligatures came away on the same day; that of Mr. Cooper on the twelfth; and that of Dr. Sykes on the tenth day.

In the six cases in which only one ligature was used, it came away on the thirty-ninth day, in one on the eleventh, in one on the twenty-first, and in two the day is not mentioned.

The operation of Brasdor, of applying the ligature to the distal side of the tumour, was performed in six of the cases.

Of these, five were females, and three, or just one-half, terminated fatally.

In case number two, the patient died three months after the operation, of hypertrophy of the heart, and it is likely this was the real cause of her death; at any rate it was a very unfavorable case for an operation of any kind.

The patient mentioned in case four died two months after the operation from repeated hemorrhages, and in case five ulceration of the wound with hemorrhage destroyed the patient eleven days after the operation.

In four of these cases the right carotid was affected; the left in the remaining two.

The occupation of the patients is not given in a single case.

The cause of the disease is mentioned in but three instances. In one, the patient referred it to palpitation of the heart from domestic affliction; in another the patient supposed it to be owing to the night air; and in a third it was referred to severe squeezing. Prior to the deligation of the artery, the disease had existed in these patients from eleven days to three years.

In case one, it was perceived eleven days previous to the operation; in one six months, in one seventeen months, in one several months, in one one month, and in one three years.

The volume of the tumour is noticed in all the cases but one. In three it was very large, in one two inches in diameter by two in breadth, and in one the size of a pullet's egg.

*Summary.*—The foregoing seventeen cases are all I am able to find in the medical and surgical works which I have examined, and these are by no means few in number. Aneurism of the carotid is generally believed to occur oftener in males than females, agreeing in this respect with that of the different arteries, but in the cases which I have reported, it appears that females were affected in ten instances, while it occurred in only seven males.

The ages of the patients were as follows. In three it occurred between ten and twenty, in two between twenty and thirty, in two between thirty and forty, in three between forty and fifty, in two between fifty and sixty, in two between sixty and seventy, in one between seventy and eighty.

The artery of the left side was affected in five of the cases, the right in ten, in one it occurred on both sides (though the artery of but one side was tied), and in one it is not mentioned.

The occupation of the patients is mentioned in five of the cases only. Of these, there were two laborers, one a porter, one a huntsman, and one a prostitute.

The cause of the disease is stated in only eight instances. In these, it occurred, in two, from wounds, in two it was caused by a fall from a horse, in one from exposure to night air, in one from domestic affliction; it was referred to severe squeezing in one, and in one it was caused by straining the neck.

The existence of the disease, prior to the treatment, is stated in all the cases, except four; varying from eleven days to three years. It was as follows: in two it had existed eleven days, in two one month, in one several months, in one three and a half months, in two six months, in one nine months, in one eleven months, in one seventeen months, and in two three years.

Of the fourteen cases in which the volume of the aneurismal tumour is mentioned it was as follows:

In one it was the size of a hen's egg, in one of a woman's two fists, in one of the volume of an egg, in one of the size of a goose egg, in one of a pullets egg, in one of a walnut, in one of a large walnut, in five very large, in one two and a half inches in breadth, in one five inches in length by four in depth.

The length of time required in performing the operation is noticed only in one case, that of Mr. Montgomery, in which it occupied twenty-five minutes.

In one of the cases cured by Valsalva's method, the patient was under treatment about eighteen months, while the other was considered as cured in seven months.

In the cases in which one ligature was used, it was cast off, in one, on the eleventh day, in one on the seventeenth, in one on the ninetenth, in one on the twenty-eighth, in one on the thirty-ninth, and in one on the twenty-first. In those in which two ligatures were employed, they separated, in one, on the twelfth day, in one on the tenth, in one the upper came away on the fifteenth, and the lower on the seventeenth, and in six the day is not noticed.

Six, or rather more than one-third, terminated fatally; of these death occurred, in one, on the third day after the operation, in one on the twenty-first, in one on the sixty-seventh, in one in two months, in one in three, and in one in four months.

The cause of death, in two, was inflammation of the sac, hypertrophy of the heart in one, repeated hemorrhage in one, in another death occurred from ulceration and hemorrhage, and in one no dissection is given.

